# Peculiar Odorous Principle in the Blood of Man

**Published:** 1829-10-01

**Authors:** 


### XXIV.

### Peculiar Odorous Principle in the Blood of Man, distinguishing it from that of other Animals.

Such is the title of a Memoir published in the Annals of Public Health and Legal Medicine, by M. Barruel, "chef des Travaux Cliniques," to the Faculty of Medicine. He observes, that a medico-legal question not unfrequently arises respecting stains from blood on clothes. The chemist may pronounce that a stain is produced by blood, and not by any other colouring matter, but he cannot by possibility pretend to affirm whether that blood be or be not human. Our author had long been engaged in obtaining the colouring matter from the blood, by boiling that of bulls, coagulated with a great excess of tolerably concentrated sulphuric acid. In conducting the experiments he was constantly struck with the strong smell of the stall *(odeur de bouverie)* emitted, but thought very little on the subject, till an accident occurred that drew his attention more particularly to the inquiry.

An individual, after losing considerably at play, swallowed a large quantity of opium, with the view of self-destruction. M. Orfila was summoned, and as a copious bleeding had been practised, resolved to ascertain whether any traces of morphine existed in the blood. M. Barruel assisted, and the blood having first been coagulated in a *bain-marie*, it was triturated, without affording any peculiar odour. It was then boiled with a sufficient quantity of sulphuric acid and water, when there issued from the flask so intolerable an odour of human perspiration, that the operator was fairly obliged to make good his retreat for some seconds from the laboratory. It now struck our author that it might perhaps be possible to distinguish the human blood by this test from that of other animals, and accordingly he set to work, and ultimately arrived at the following conclusions.

1mo. The blood of each species of animal contains a principle peculiar to itself.

2do. This principle, which is highly volatile, has an odour like that of the perspiration, or cutaneous and pulmonary exhalation of the animal from which the blood is obtained.

3tio. This volatile principle is combined with the blood and not to be perceived as long as the combination obtains.

4to. When the combination ceases the odorous principle of the blood is volatilized, and it becomes not only possible, but absolutely easy to recognise the animal that furnished it.

5to. In every kind of animal this odorous principle is more intense in the male than in the female; and, moreover, the colour of the hair in the individual, is attended with differences in the odour.

6to. This principle is dissolved in the blood, so that it may be shewn not only in the blood, as a whole, but when deprived of fibrine, or in the serosity.

7mo. Of all the modes employed to liberate the principle in question, the sulphuric acid is the best.

In order to obtain the foregoing results, it is only necessary to pour some drops of blood, or serum into a glass, and throw on it a slight excess of concentrated sulphuric acid (about a third or half the volume of the blood,) and stir it with a glass rod. The odour that arises, as was mentioned before, always resembles the sweat of the animal that supplies the blood. Detailed statements are appended respecting the odour from the blood of oxen, horses, sheep, &c. but as the above rule states the general fact, we need not particularize each animal in rotation. It became important to ascertain whether dried stains of blood upon linen would furnish any indication, and M. Barruel asserts, that provided the stain be of a certain size, it is easy to recognize the kind of blood, even after the lapse of more than fifteen days. The portion of linen stained must be cut off, placed in a glass vessel, and allowed to lie quietly in a small quantity of water for some time. When the spot is well soaked, the sulphuric acid is poured on it, and stirred with a glass rod as before. Our author is ignorant if after a greater length of time than 15 days, satisfactory results could be obtained.

M. Barruel concludes with some hints to the Juges d'Instruction, and earnest recommendations to the profession to regale their olfactory organs. If the present become an established mode of legal diagnosis, "smelling a savoury suit," will no longer be a figurative expression! Seriously, however, the facts above detailed are curious, if authentic An experiment too to determine their accuracy would be easily made.

